# Identification of CD73 as the Antigen of an Antigen-Unknown Monoclonal Antibody Established by Exosome Immunization, and Its Antibody–Drug Conjugate Exerts an Antitumor Effect on Glioblastoma Cell Lines

**DOI:** 10.3390/ph15070837

**Published:** 2022-07-06

**Authors:** Takahiro Anzai, Shinji Saijou, Hiroki Takashima, Misato Hara, Shingo Hanaoka, Yasuhiro Matsumura, Masahiro Yasunaga

**Affiliations:** 1 Division of Developmental Therapeutics, Exploratory Oncology Research & Clinical Trial Center, National Cancer Center, Chiba 277-8577, Japan; taanzai@east.ncc.go.jp (T.A.); ssaijou@east.ncc.go.jp (S.S.); hitakash@east.ncc.go.jp (H.T.); shanaoka@east.ncc.go.jp (S.H.); yhmatsum@ncc.go.jp (Y.M.); 2 Research Division, RIN Institute, Inc., Tokyo 104-0045, Japan; 3 Tamagawa Seiki Co., Ltd., Nagano 395-8515, Japan; misato-hara@tamagawa-seiki.co.jp; 4 Department of Immune Medicine, National Cancer Center Research Institute, National Cancer Center, Tokyo 104-0045, Japan

**Keywords:** monoclonal antibody, CD73, exosome, antibody–drug conjugate, glioblastoma

## Abstract

Development of antibodies against the native structure of membrane proteins with multiple transmembrane domains is challenging because it is difficult to prepare antigens with native structures. Previously, we successfully developed a monoclonal antibody against multi-pass membrane protein TMEM180 by exosome immunization in rats. This approach yielded antibodies that recognized cancer-specific antigens on the exosome. In this study, we performed immunoprecipitation using magnetic beads to identify the antigen of one of the rat antibody clones, 0614, as CD73. We then converted antibody 0614 to human chimeric antibody 0614-5. Glioblastoma (GB) was the cancer type with the highest expression of CD73 in the tumor relative to healthy tissue. An antibody–drug conjugate (ADC) of 0614-5 exerted an antitumor effect on GB cell lines according to expression of CD73. The 0614-5-ADC has potential to be used to treat cancers with high CD73 expression. In addition, our strategy could be used to determine the antigen of any antibody produced by exosome immunization, which may allow the antibody to advance to new antibody therapies.

## 1. Introduction

There is considerable motivation to identify new target molecules for the diagnosis and development of new cancer drugs. Among these new target molecules, the development of antibodies against the native structure of membrane proteins with multiple transmembrane domains is challenging because of the difficulty in preparing antigens with the native structure. Immunizing mice or rats directly with whole cells expressing membrane protein as antigens is one method of solving this problem [1,2]. However, because cells express many other membrane proteins, direct immunization of the entire cell also produces many antibodies to antigens other than the target antigen. In previous work, we identified a new colorectal cancer tumor marker multi-pass membrane protein, TMEM180, which is also secreted in exosomes [3]. The discovery that TMEM180 is present on exosomes inspired us to immunize concentrated TMEM180-positive exosomes from cancer cells, which have fewer components than whole cancer cells, in order to increase antigenicity. We prepared TMEM180-positive exosomes from the supernatant of a serum-free culture medium of colorectal cancer cell DLD-1.

We succeeded in developing an anti-TMEM180 monoclonal antibody by immunizing exosomes in rats as expected [3], but because exosomes from cancer cells still contain different membrane proteins, we also established antibody clones that recognize each unknown antigen with high cancer specificity. If each of these antigens can be determined, it will lead to antibodies to membrane proteins that are difficult to prepare. There are several successful methods for determining the antigens, for example, mass purification of antigens from antibody-positive cells and confirmation by Western blotting [4], the introduction of cDNA libraries into the cells, and then identification of antigens by enrichment of antibody-positive cells [5]. However, there is no general method to search for and determine such antigens, a situation that requires a lot of trial and error.

In this study, we identified the antigen of a cancer-specific recognition monoclonal antibody as CD73 by immunoprecipitation of exosomes using magnetic beads with reduced nonspecific binding and characterized the antibody. We also identified glioblastoma (GB) as cancer with the highest cancer-specific expression of CD73 and demonstrated that an antibody–drug conjugate of the anti-CD73 monoclonal antibody exerted an antitumor effect on our GB cell lines.

## 2. Results and Discussions

### 2.1. Identification of CD73 as the Target Antigen of Antibody Clone 0614

**Figure 1:**
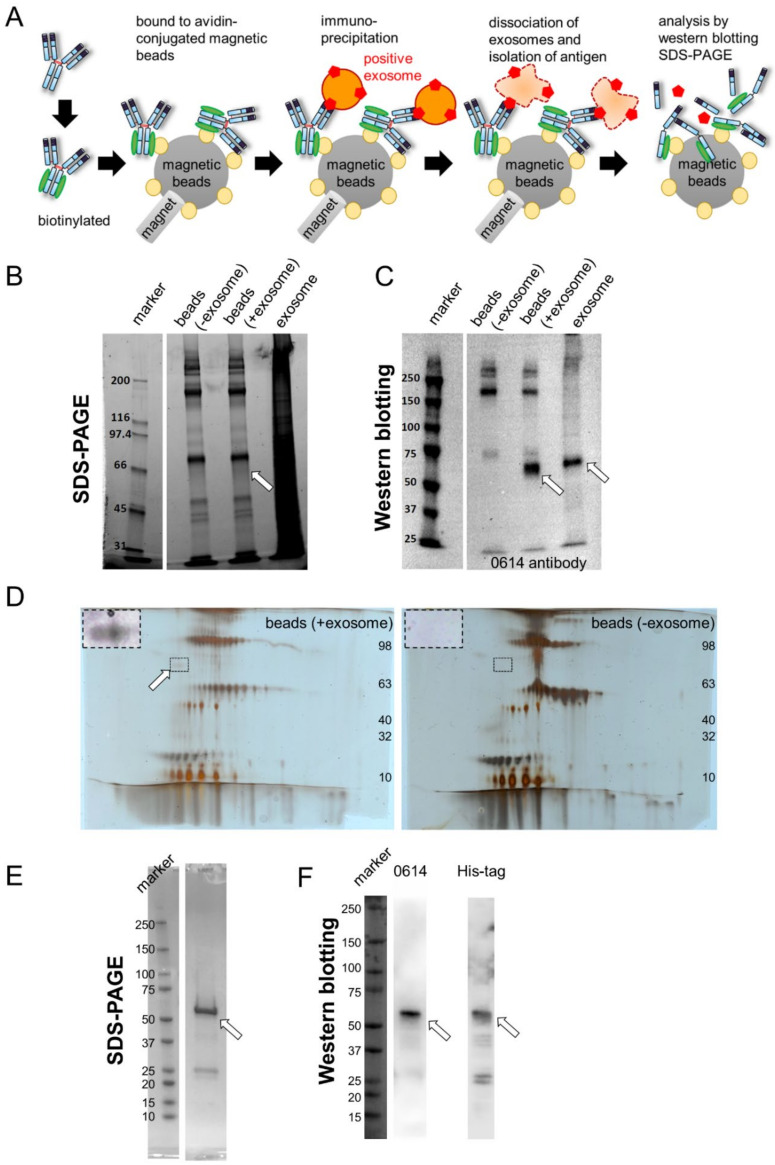
Identification of CD73 as the antigen of antibody 0614. (**A**) Schema for identification of the unknown antigen of antibody 0614. (**B**,**C**) SDS-PAGE (**B**) and Western blot (**C**) analyses of samples immunoprecipitated with avidin magnetic beads conjugated to biotinylated antibody 0614. Target antigen containing exosome samples was loaded as a positive control. The position of the target antigen is indicated by a white arrow. (**D**) 2D-PAGE analysis of samples immunoprecipitated with avidin magnetic beads conjugated to biotinylated antibody 0614. The position of the target antigen is indicated by a white arrow. The part surrounded by the broken line has been adjusted for contrast and enlarged in the upper left of the figure. (**E**,**F**) SDS-PAGE (**E**) and Western blot (**F**) analyses of recombinant CD73 fused to the His-tag. Western blot analyses were performed using antibody 0614 (**left**) and anti–His tag antibody (**right**).

We developed antibodies by exosome immunization, as described previously [3]. Briefly, we immunized rats with tumor-derived exosomes purified from the supernatant of the colon cancer cell line DLD-1. We then analyzed supernatants of hybridoma cells containing antibodies by flow cytometry and screened both DLD-1 colorectal cancer (CRC) cells (positive) and K562 myeloma cells (negative) (Figure S1, Supplementary Materials). We established a rat IgG2a clone, 0614, as an antibody specific to unknown antigens. To identify the antigen, we isolated and purified it from exosomes (Figure 1A). In this approach, the target antibody is first biotinylated and bound to avidin-conjugated magnetic beads. Then, these beads are bound to an antigen-positive exosome. An exosome–antibody complex is isolated by immunoprecipitation. After the antigen is dissociated from magnetic beads, it is detected by SDS-PAGE and Western blotting (Figure 1A). We then performed immunoprecipitation of exosomes derived from DLD-1 cells using biotinylated antibody 0614 and avidin-bound magnetic beads, and analyzed the resultant samples using SDS-PAGE (Figure 1B) and Western blotting (Figure 1C). We succeeded in obtaining an antigen candidate of about 70 kDa. To allow more specific antigen determination, the samples were separated by two-dimensional electrophoresis (Figure 1D). In the same region at 50–75 kDa, we selected the spot that was observed in the exosome-positive samples but not in the exosome-negative samples (Figure 1D, surrounded by the broken line).

Mass spectrometry revealed 5′-nucleotidase (UniProt:P21589) and heat shock 70 kDa protein 1A (UniProt:P0DMV8) and 1B (UniProt:P0DMV9) as candidates. Because the antigen of 0614 can be detected by flow cytometry, we focused on 5′-nucleotidase, also called CD73. We constructed and purified the extracellular domain of CD73 [6,7,8] with a C-terminal His-tag as a recombinant protein (Figure 1E), and then performed Western blotting; this analysis confirmed that the antigen of antibody 0614 is CD73 (Figure 1F). In functional terms, CD73, an enzyme that converts AMP to inorganic phosphate and adenosine, localizes to the membrane surface of regulatory T cells and cancer cells and mediates T-cell immunosuppression [9,10]. There is a report that inhibitory antibodies targeting CD39 (an enzyme that converts ATP to AMP) and CD73 inhibit the adenosine pathway and promote activation of the immune system against cancer [11]. There are some reports that CD73 expressed on exosomes has enzymatic activity, and exosome-derived adenosine results in T-cell inhibition and impairs the antitumor immune response [12,13,14]. Thus, as it is reasonable that an anti-CD73 antibody could be generated by exosome immunization, we decided to proceed with development of a 0614 antibody that recognizes CD73 on exosomes.

### 2.2. Characterization of Chimeric Antibody Clone 0614-5

**Figure 2:**
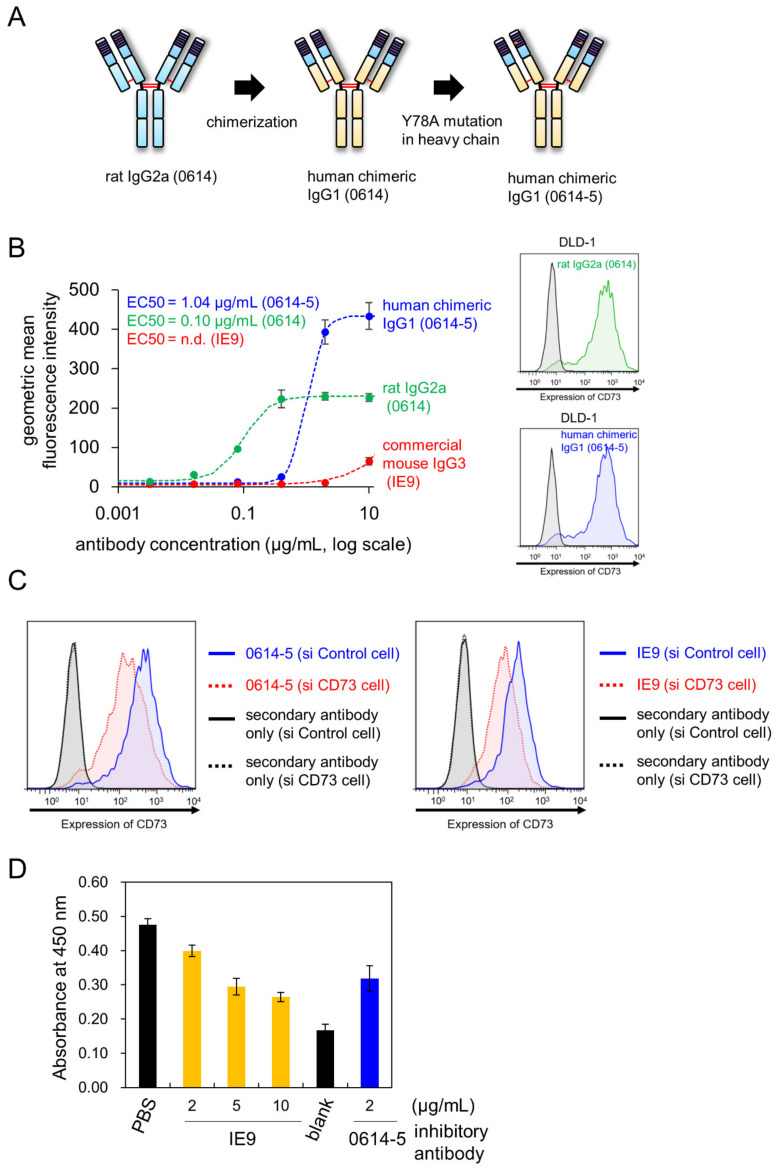
Characterization of human chimeric monoclonal antibody 0614-5. (**A**) Schema for generating human chimeric antibody of rat 0614, 0614-5. (**B**, **left**) Flow cytometry data of serially titrated rat 0614 mAb (green), human chimeric 0614-5 mAb (blue), and commercially available anti-CD73 mAb IE9 for DLD-1 cells. Dashed lines show the fitting curve calculated by ImageJ1.52 (using Rodbard sigmoid fitting). *N* = 3. Data are shown as means ± standard deviation. n.d. = no data. (**B**, **right**) Flowcytometry analysis of rat 0614 mAb (green, 1 µg/mL) and human chimeric 0614-5 mAb (blue, 1 µg/mL). (**C**) Flowcytometry analysis of human chimeric 0614-5 mAb (1 µg/mL) and anti-CD73 mAb (IE9, 3 µg/mL) using CD73 gene knockdown DLD-1 cells (shown in red) by siRNA. (**D**) Sandwich ELISA analysis of 0614-5 mAb and IE9 mAb monitored by His-tagged CD73. 0614-5 was used as a capture antibody in this figure. The results for blocking by IE9 mAb are shown in orange, and the results for blocking by 0614-5 mAb (positive control) are shown in blue. PBS was used as a nonblocking negative control. Blank indicates a condition that does not contain CD73. *N* = 3. Data are shown as means ± standard deviation.

We converted the rat IgG2a antibody to human chimera IgG1 in order to evaluate its potential therapeutic applications (Figure 2A). During construction of the chimeric antibody, we found that the Y78A mutation in the heavy chain reduced nonspecific binding in immunohistochemistry experiments (data not shown). The impacts of mutations in this antibody will be described in detail elsewhere. We designated the human chimeric mAb (monoclonal antibody) 0614-5 (Figure 2A). Next, we compared the affinities of 0614 antibody (rat IgG2a), 0614-5 chimeric antibody (human IgG1), and commercial antibody IE9 (mouse IgG3) to DLD-1 cells. Although an exact comparison was difficult due to the different secondary antibodies detected in each, the EC50 for rat IgG2a 0614 was 0.10 µg/mL, the EC50 for human IgG1 0614-5 was 1.04 µg/mL, and no data were available for mouse IgG3 IE9 (Figure 2B). To further confirm that there was no change in specificity for CD73, CD73 gene knockdown DLD-1 cells were generated by siRNA, and flow cytometry analysis was performed. Analysis using a commercially available anti-CD73 antibody IE9 clone as the positive control showed that the specificity of the chimeric antibody 0614-5 for CD73 was maintained (Figure 2C).

To investigate whether this antibody had neutralizing activity, a CD73 activity inhibition assay was conducted. We could not obtain anti-CD73 antibodies in clinical trials known to inhibit CD73 enzymatic activity (for example, AK119, BMS-986179, CPI-006, MEDI9447, NZV930, Sym024, and TJ004309) [15] as a positive control. Therefore, we reviewed the literature and found that of the commercially available clones, IE9 is an antibody that inhibits CD73 enzymatic activity [16,17]. Inhibition experiments were performed by increasing the antibody concentration up to 30 µg/mL, but neither IE9 nor 0614-5 showed inhibitory activity at this concentration (data not shown). Therefore, to ascertain whether the IE9 and 0614-5 epitopes are in the vicinity of CD73, competitive inhibition experiments were performed by sandwich ELISA (enzyme-linked immunosorbent assays). First, IE9 was used as a capture antibody, but binding to CD73 could not be detected (data not shown). Next, 0614-5 was used as a capture antibody, and IE9 antibody was added at varying concentrations, followed by the addition of His-tagged recombinant CD73 as an antigen for a competitive reaction. After washing, a residual antigen was detected with an anti-His tag antibody. The results show that a competitive reaction occurred as the concentration of IE9 antibody was increased (Figure 2D). This indicates that the IE9 and 0614-5 epitopes are in close proximity to CD73. 0614-5 may also have enzyme inhibitory activity, as does IE9. In future work, we will determine the epitope of 0614-5 and confirm if it can inhibit the enzymatic activity of CD73.

### 2.3. Choosing Cancer Types for Targeting by 0614-5 mAb

**Figure 3:**
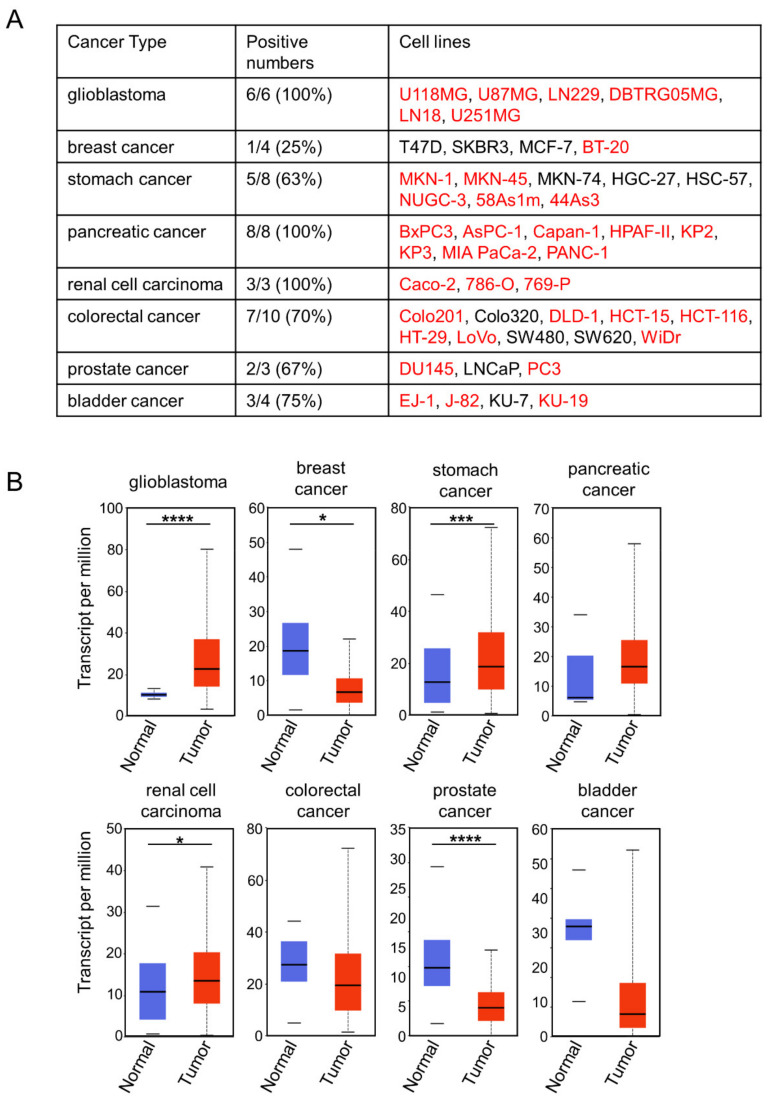
CD73 has the highest cancer specificity for glioblastoma. (**A**) Comparison of CD73 expression in cell lines from different cancer types (glioblastoma, breast cancer, stomach cancer, pancreatic cancer, renal cell carcinoma, colorectal cancer, prostate cancer, and bladder cancer), as determined by flow cytometry analysis. Names of positive cell lines are shown in red. (**B**) CD73 mRNA expression in different cancer types (glioblastoma, breast cancer, stomach cancer, pancreatic cancer, renal cell carcinoma, colorectal cancer, prostate cancer, and bladder cancer), comparing tumor (shown in red) with normal tissue (shown in blue). * *p* < 0.05, *** *p* < 0.001, **** *p* < 0.0001.

To determine the ideal clinical application of 0614-5 mAb, we screened for cancer types with high rates of CD73 positivity: either high expression in cancer cell lines or high levels in cancer tissue relative to normal samples from patients. Comparison of CD73 expression in glioblastoma, breast cancer, stomach cancer, pancreatic cancer, renal cell carcinoma, colorectal cancer, prostate cancer, and bladder cancer by flow cytometry revealed that glioblastoma (6/6), pancreatic cancer (8/8), and renal cell carcinoma (3/3) cell lines were 100% CD73-positive (Figure 3A), and immunostaining data from the human protein atlas revealed that glioma (11/12), pancreatic cancer (11/12), and colorectal cancer (12/12) were 91.6%, 91.6%, and 100% CD73-positive (Figure S2, Supplementary Materials). Next, we compared CD73 gene expression in these cancer types using UALCAN [18], based on The Cancer Genome Atlas (TCGA) genomics data from tumor and normal samples. Among the eight cancer types, glioblastoma exhibited the highest expression of CD73 in the tumor vs. normal sample (Figure 3B). GB is among the most malignant brain tumors and is the deadliest type of cancer. Various treatments for GB are being developed, including antibody therapy [19]. CD73 is a negative prognostic factor for GB [20]. Based on these results, we decided to target GB with 0614-5 mAb.

### 2.4. Application of Clone 0614-5 Antibody–Drug Conjugate in Glioblastoma Cell Lines

**Figure 4:**
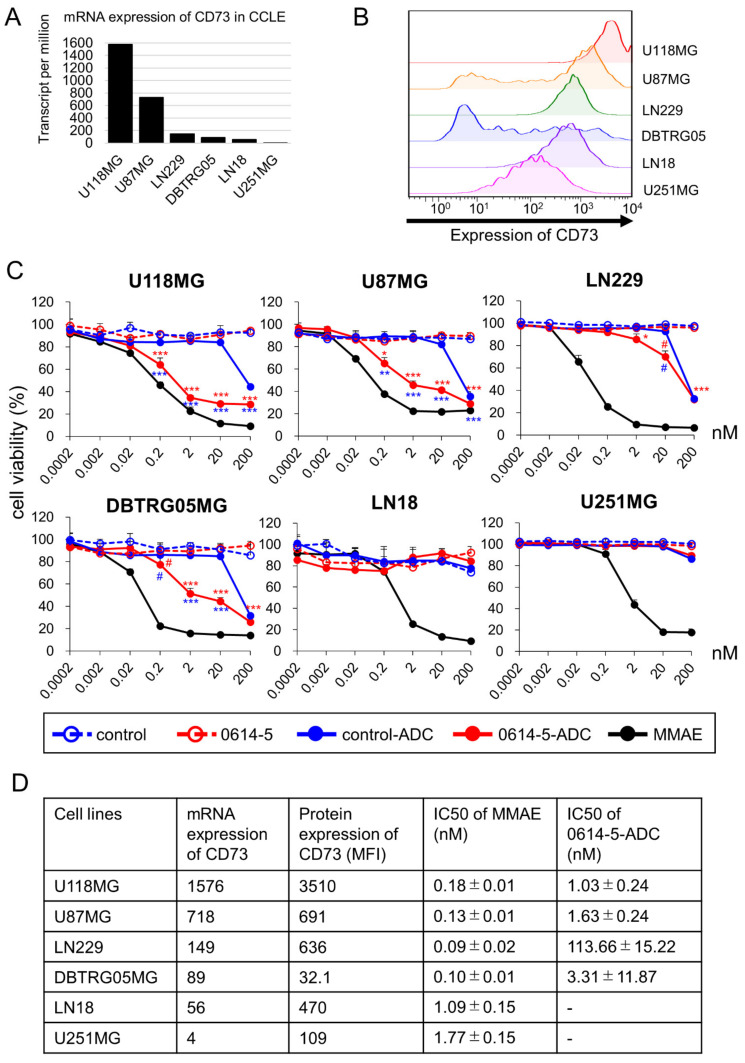
0614-5-ADC exhibits antitumor effects in GB cell lines depending on the expression level of CD73. (**A**) mRNA expression of CD73 in GB cell lines (U118MG, U87MG, LN229, DBTRG05MG, LN18, and U251MG). (**B**) Flow cytometry analysis of CD73 in GB cell lines (U118MG, U87MG, LN229, DBTRG05MG, LN18, and U251MG) (**C**) Cytotoxicity assays performed on the GB cell lines shown in (**A**,**B**). *N* = 3. Data are shown as means ± standard deviation. ^#^ *p* < 0.05, * *p* < 0.01, ** *p* < 0.001, *** *p* < 0.0001 (red symbol: 0614-5-ADC vs. 0614-5, blue symbol: 0614-5-ADC vs. control-ADC). (**D**) Summary of CD73 expression in (**A**,**B**) and IC50 against GB cell lines of 0614-5-ADC or MMAE in Figure 4C.

The enzyme inhibitory activity of the 0614-5 antibody remains unknown, but no direct cytotoxicity in GB cells has been observed (data not shown, Figure 4C). Thus, we investigated the usefulness of our 0614-5 antibody as an ADC. In our in vitro cytotoxicity assays, we used six GB cell lines (U118MG, U87MG, LN229, DBTRG05MG, LN18, and U251MG). When we examined the expression of the CD73 gene in each cell line using a Cancer Cell Line Encyclopedia (CCLE) database [21], the order was as shown in Figure 4A. We also analyzed the expression of CD73 on the cell membrane by flow cytometry (Figure 4B and Figure S3, Supplementary Materials). Gene expression and protein expression of CD73 were well-correlated. We prepared 0614-5 mAb and control mAb conjugated with MMAE (0614-5-ADC and control-ADC). There was no significant difference in binding activity between 0614-5 and 0614-5-ADC (Figure S4, Supplementary Materials). Then, we assessed in vitro cytotoxicity of the control naked antibody, 0614-5 naked antibody, control-ADC, 0614-5-ADC, and free linker MMAE against GB cell lines. 0614-5-ADC showed statistically significant differences in cytotoxicity than the control-ADC in four cell lines (U118MG, U87MG, LN229, and DBTRG05MG), and the 0614-5 naked antibody had no toxic effect (Figure 4C). In U251MG cells, which were weak for CD73 expression, 0614-5-ADC exerted no cytotoxic activity (Figure 4C). Despite similar levels of CD73 gene expression, DBTRG05MG exhibited ADC-induced cytotoxicity, whereas LN18 did not (Figure 4A,C). Possible reasons for these situations include U251MG and LN18 being about 10 times less sensitive to MMAE than the other four cell lines (Figure 4C,D) and protein expression being highly heterogeneous in DBTRG05MG (Figure 4B and Figure S3, Supplementary Materials). Together, these results show that our ADC exerted cytotoxicity depending on the expression level of CD73. In this in vitro system, 0614-5-ADC had a lower cell-killing effect than free MMAE (Figure 4C,D), but such ADCs were markedly accumulated in solid tumors due to the EPR effect in vivo as compared with free low-molecular drug. It is, therefore, expected that the in vivo antitumor effect of the ADC will be significantly higher than that of free MMAE [22,23,24].

Currently, several anti-CD73 antibodies are in clinical trials (for example, AK119, BMS-986179, CPI-006, MEDI9447, NZV930, Sym024, and TJ004309) [15], and all of these antibodies block the enzymatic activity of CD73 and are used as naked antibodies. ADCs are powerful drug delivery tools that can deliver chemotherapeutic agents specifically to target tumor cells [25]. There are few examples of anti-CD73-ADC development other than reports for small cell lung cancer [26]. Our report also demonstrates the utility of anti-CD73-ADCs as potential new therapeutic candidates for GB. A limitation of this paper is that it is not directly comparable to our established 0614 antibody because of the difficulty of obtaining anti-CD73 antibodies with superior efficacy that have already progressed to clinical trials, as mentioned earlier. We hope that future findings, including in vivo experiments, and comparisons with other clones, will demonstrate that 0614-5-ADC may be a useful antibody drug candidate for the development of CD73-targeted therapies.

## 3. Materials and Methods

### 3.1. Cells and Cell Culture

DLD-1, K562, U118MG, U87MG, LN229, DBTRG05MG, LN18, U251MG, and the cell lines listed in Figure 3A were purchased from American Type Culture Collection or the Japanese Collection of Research Bioresources Cell Bank. These cell lines were cultured at 37 °C under a 5% CO_2_ atmosphere in DMEM (FUJIFILM Wako, Osaka, Japan) or RPMI-1640 (FUJIFILM Wako) supplemented with 10% FBS (Thermo Fisher Scientific, Waltham, MA, USA) and 1% penicillin–streptomycin–amphotericin B suspension (FUJIFILM Wako). For generating gene knockdown cells, DLD-1 cells were plated in 6-well plates and CD73 custom siRNA (5′-GCCACUAGCAUCUCAAAUA-3′, Merck, Tokyo, Japan) or control siRNA (Dharmacon, Boulder, CO, USA) were transfected using Lipofectamine RNAiMAX (Thermo Fisher Scientific) according to the manufacturer’s instructions and cultured at 37 °C under a 5% CO_2_ atmosphere for 48 h.

### 3.2. Plasmid Construction

To generate chimeric antibodies, cDNAs encoding the heavy-chain variable region and kappa light-chain variable region of antibody 0614 were PCR-amplified using the following primers: heavy chain, 5′-GGATCCAACCCTTCGAATTCCACCATGGACATCAGGCTCAGC-3′ and 5′-GATGGGCCCTTGGTGCTAGCTGAGGAGACTGTGAGCATGACT-3′; light chain, 5′-GGATCGAACCCTTCGAATTCCACCATGATGGCTCCAGTTCAA-3′, and 5′-GATGGTGCAGCCACCGTACGTTTCAATTCCAGCTTGGTGCCT-3′. PCR products were cloned into vector pcDNA3.3 (Thermo Fisher Scientific) for human IgG1 expression using In-Fusion cloning technology (Takara Bio, Shiga, Japan) as described previously [3]. For generation of 0614-5, antibody constructs were generated by inverse PCR using primers 5′-AGCACTGCCTATCCAGACTCTGTGAAG-3′ and 5′-TGGATAGGCAGTGCTACCACCAGTATT-3′ against the 0614 heavy-chain expression vector. The soluble domain (residues 27–549) of the human CD73 gene was PCR-amplified from cDNA from DLD-1 cells with an NdeI restriction site in the 5′ region and a XhoI restriction site in the 3′ region, using primers 5′-AGAAGGAGATATACATATGTGGGAGCTTACGATTTTG-3′ and 5′-GTGGTGGTGGTGGTGCTCGAGGGAAAACTTGATCCGA-3′. The PCR products were cloned into pET21a (Novagen, Madison, WI, USA) using In-Fusion cloning technology. All plasmid constructs used in this study were verified by DNA sequencing.

### 3.3. Antibodies and Recombinant Proteins

Rat antibody 0614 was generated by exosome immunization as described previously [3]. HRP-linked 0614 rat antibody was prepared using the Peroxidase Labeling Kit-NH2 (Dojindo, Kumamoto, Japan). Chimeric antibody expression was performed using the ExpiCHO Expression System (Thermo Fisher Scientific). Culture supernatants were purified using rProteinA Sepharose fast flow (GE Healthcare, Chicago, IL, USA) and Superdex 200 16/600 column (GE Healthcare). Purified monoclonal antibodies were buffer-exchanged into PBS, concentrated using Vivaspin Turbo 15 30-kDa centrifugal filter units (Sartorius, Göttingen, Germany), and stored at 4 °C until use. Recombinant CD73 protein was overexpressed in E. coli Rosetta (DE3) cells. The insoluble fraction was solubilized and purified by Ni-NTA agarose resin (Invitrogen, Carlsbad, CA, USA) under denaturing conditions (8 M urea). Purified denatured CD73 was refolded by the rapid dilution method using a refolding buffer consisting of 20 mM Tris HCl pH 8.0, 0.5 M l arginine, and 10% glycerol. Renatured CD73 protein was purified by Superdex 75 16/600 column. Purified CD73 was buffer-exchanged into PBS, concentrated using Amicon ultra 4 10-kDa centrifugal filter units (Merck Millipore, Burlington, MA, USA), and stored at 4 °C until use. Purity of proteins was determined by SDS-PAGE using 4–15% Mini-PROTEAN TGX gel (Bio-Rad, Hercules, CA, USA). The C-terminal purification His-tag was not removed.

### 3.4. Isolation of Antigen of 0614 Antibody from Exosome Sample

0614 antibodies were biotinylated with EZ-Link NHS-LC-Biotin (Thermo Fisher Scientific). Biotinylated antibody 0614 (10 µg) and 0.1 mg of streptavidin-conjugated magnetic beads (FG beads, Tamagawa Seiki, Nagano, Japan) were reacted at 4 °C for 1 h, and then washed by magnetic separation. Exosomes from CRC lines or exosomes from blood cell lines (as a negative control) were reacted for 2 h with 0.1 mg of antibody 0614-conjugated magnetic beads in PBS-T. To crosslink antibodies and antigens, 5 nmol of DTSSP was reacted at room temperature for 30 min, and then the reaction was quenched with 50 nmol of Tris for 15 min. Sample collected by magnetic beads was solubilized in RIPA buffer (FUJIFILM Wako) and then analyzed by SDS-PAGE and Western blotting. SDS-PAGE was performed using 7.5% Mini-PROTEAN TGX gel (Bio-Rad), and the gels were stained with Oriole fluorescent gel stain (Bio-Rad). For Western blotting, samples were transferred to PVDF membranes (Merck Millipore), and the membranes were blocked for 15 min with StartingBlock (TBS) Blocking Buffer (Thermo Fisher Scientific). The blocked membranes were washed three times with TBS with 0.1% Tween 20 (TBS-T) and then incubated with HRP-linked antibody 0614 (2 µg/mL) at room temperature for 1 h. After the membranes were washed five times with TBS-T, the proteins were visualized using Chemi-Lumi One Super (Nacalai Tesque, Kyoto, Japan).

### 3.5. Identification of Antigen of 0614 Antibody

For 2D-PAGE, acetone precipitation was performed, and 20 µL samples were mixed with 80 µL of ice-cold acetone and stored at −20 °C for 3 h. The samples were then centrifuged at 20,000× *g* for 10 min at 4 °C, and after the supernatant was discarded, the sediment was dried. The samples were resuspended with 15 µL of 1% n-octyl-β-d-glucoside/8 M urea/2 M Thiourea and centrifuged at 20,000× *g* for 5 min at 4 °C. Supernatants (10 µL) were subjected to 2D-PAGE by Auto2D (SHARP, Osaka, Japan). Gel staining was performed using the Silver Stain MS Kit (FUJIFILM Wako). Shotgun proteome analysis was performed by Medical ProteoScope Co., Ltd. Target protein spots and control spots were excised from the gel and enzymatically digested in-gel as previously described [27]. Gel pieces were destained using the Silver Stain MS Kit (FUJIFILM Wako) and washed with acetonitrile. Samples were alkylated with DTT and iodoacetamide, and then trypsinized for 16 h at 37 °C. Trypsin-digested peptides were extracted with 25 mM ammonium hydrogen carbonate, 5% formic acid, and acetonitrile and then dried. Liquid chromatography was performed on a Paradigm MS4 (Michrom Bioresources, Auburn, CA, USA) consisting of a capillary separation column with reversed-phase L-C18 gels (l-column Micro, 0.1 mm × 15 cm, Chemicals Evaluation and Research Institute, Tokyo, Japan). Solvent A (98% distilled water with 2% acetonitrile and 0.1% formic acid) and Solvent B (5% distilled water with 95% acetonitrile and 0.1% formic acid) were used with a linear gradient made from 5 to 35% solvent B for 20 min and then 35% to 90% B for 5 min, maintained at 90% B for 5 min, then from 90% B to 5% B for 0.01 min, followed by re-equilibration with 5% B for 10 min at a flow rate of 500 nL/min. MS/MS analysis was performed on a LTQ Orbitrap XL (Thermo Fisher Scientific). The LTQ was operated in the data-dependent MS/MS mode to automatically acquire up to three successive MS/MS scans in the centroid mode. The top 10 intense precursor ions for these MS/MS scans could be selected from a high-resolution MS spectrum (survey scan) previously acquired by the Orbitrap during a predefined short time window in the profile mode at a resolution of 30,000 in the *m*/*z* range of 300 to 1500. Data were analyzed using Mascot v2.5.1 (Matrix Science, London, UK), and an in-house database.

### 3.6. Confirmation of Antigen of 0614 Antibody as CD73

The antigen of antibody 0614 was confirmed by Western blotting. Recombinant CD73 with a C-terminal His-tag was loaded onto SDS-PAGE gel, separated, and then transferred to PVDF membranes (Bio-Rad). Membranes were blocked for 5 min with Bullet Blocking One for Western blotting (Nacalai Tesque). The membranes were washed three times with TBS-T. The blots were then incubated with antibody 0614 (10 µg/mL) or anti-His tag antibody (1:10,000, ProteinTech, Rosemont, IL, USA) at room temperature for 1 h. The membranes were washed three times with TBS-T and then incubated with HRP-conjugated anti-rat IgG (1:5000; Jackson Immuno Research, West Grove, PA, USA) or anti-mouse IgG (1:5000; CST, Danvers, MA, USA) at room temperature for 1 h. Can Get Signal solution (Toyobo, Osaka, Japan) was used to reduce background noise. After the membranes were washed three times with TBS-T, proteins were visualized using ECL prime (GE Healthcare).

### 3.7. Sandwich ELISA

Sandwich ELISA was performed at room temperature. MaxiSorp plates (Thermo Fisher Scientific) were coated with 100 µL of 1 µg/mL mAb diluted in PBS for 1 h. The wells were then washed with TBS-T three times and blocked with 5% (*w*/*v*) BSA/TBS-T for 1 h. After the wells were washed, 40 µL of 2, 5, or 10 µg/mL (final concentration) mAb was added to the wells, and then 10 µL of 5 µg/mL His-tagged recombinant CD73 was incubated for 1 h. After the wells were washed, anti-His-tag mAb peroxidase-conjugated (9C11, 1:5000, FUJIFILM Wako) diluted in PBS was added to the wells for 1 h. After the wells were washed, ELISA POD Substrate TMB Kit solution (Nacalai Tesque) was added and incubated for 10 min, and then the reaction was stopped by adding 2N H_2_SO_4_. The absorbance at 450 nm was recorded using a plate reader.

### 3.8. Flow Cytometry

Flow cytometry was conducted as previously described [3]. Cultured cells were stained with 0614 mAb or human chimera 0614-5 mAb or IE9 mAb as the primary antibody and Alexa Fluor 488- or 647-conjugated anti-rat or human or mouse-Ig polyclonal antibody (Thermo Fisher Scientific) as the secondary antibody. Stained cells were analyzed on a Guava easyCyte 10HT (Merck Millipore). Dead cells were stained using propidium iodide (PI) (Thermo Fisher Scientific). Data were analyzed using the FlowJo software (Tree Star, Tokyo, Japan).

### 3.9. Database Analysis

CD73 gene expression in normal and tumor samples from the TCGA cancer genomics data was analyzed using UALCAN [18]. CD73 protein expression data in different cancer types were obtained from the human protein atlas database (https://www.proteinatlas.org/ENSG00000135318-NT5E/pathology (accessed on 17 May 2022)).

### 3.10. In Vitro Cytotoxicity Assay

The drug payload MMAE was purchased from MedChem Express. Generation of ADC was conducted as described previously [28]. To conjugate to the MMAE, antibodies were incubated with 8 mM 2-MEA in a phosphate-buffered saline (PBS) solution containing 5 mM ethylenediaminetetraacetic acid (EDTA, pH 8) at room temperature for 30 min. The concentrations of the mAb conjugated with MMAE were measured using a NanoDrop. The numbers of residual thiols were analyzed with DTNB. The drug (MMAE)/antibody ratio was 4.2. Our established 372 antibody [28] that does not react with cancer cells was used as a negative control. Cells were plated at 3000 cells/well in 96-well plates and cultured overnight. After the media was replaced, the indicated concentrations of naked antibodies, ADCs, or free MMAE were added, and the sample was incubated for 72 h. Cell viabilities were monitored using the Cell Counting Kit-8 (Dojindo). Significant differences between the groups of 0614-5, 0614-5-ADC, and control-ADC were determined using ANOVA. All analyses were carried out using EZR version 1.42 [29].

## 4. Conclusions

In this study, we performed immunoprecipitation using magnetic beads to demonstrate that the antigen of rat antibody clone 0614, produced by exosome immunization, was CD73. Our strategy demonstrates that it is possible to identify the unknown antigens of useful antibodies. We also demonstrated that the human chimeric antibody 0614-5-ADC exerts antitumor effects on GB cell lines dependent on the expression of CD73. Thus, 0614-5-ADC could be used to treat cancers with high levels of CD73 expression, including GB.

## Data Availability

The raw data presented in this study are available on request from the corresponding author.

## Supplementary Materials

The following are available online at https://www.mdpi.com/article/10.3390/ph15070837/s1. Figure S1: screening of hybridoma cells containing antibodies by flow cytometry, Figure S2: CD73 enzymatic activity inhibition assay, Figure S3: CD73 protein expression summary, Figure S4: binding activity of 0614-5 and 0614-5-ADC, abbreviations list.

## Abbreviations

ADC	antibody-drug conjugate
AMP	adenosine monophosphate
ATP	adenosine triphosphate
CCLE	Cancer Cell Line Encyclopedia
CRC	colorectal cancer
EC50	Effective Concentration 50
ELISA	Enzyme-linked immunosorbent assays
EPR	Enhanced Permeability and Retention
GB	glioblastoma
IC50	Inhibitory Concentration 50
MMAE	monomethyl auristatin E
NT5E	5'-nucleotidase ecto
PI	propidium iodide
TCGA	The Cancer Genome Atlas
