# Supplementary material for: Identification of CD73 as the Antigen of an Antigen-Unknown Monoclonal Antibody Established by Exosome Immunization, and Its Antibody–Drug Conjugate Exerts an Antitumor Effect on Glioblastoma Cell Lines
Source: Pharmaceuticals (Basel). 2022 Jul 6;15(7):837. doi: 10.3390/ph15070837 (PMC9322095; doi:10.3390/ph15070837)
Supplement: Supplementary file 1 [file pharmaceuticals-15-00837-s001.zip › pharmaceuticals-1757631-supplementary.pdf]

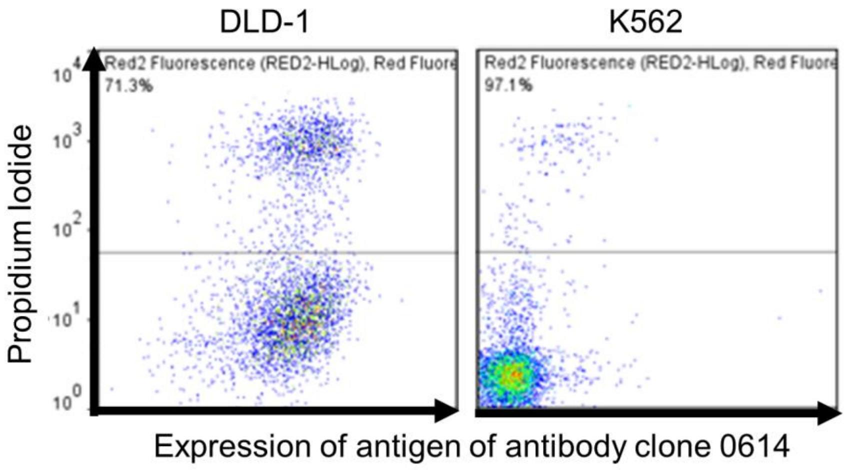

Figure S1. Screening of hybridoma cells containing antibodies by flow cytometry

Flow cytometry analysis of colorectal cancer cell line DLD-1 and myeloma cell line K562 by culture supernatant of hybridoma producing antibody 0614.

CD73 protein expression summary in The human protein atlas

Cancer Type	High or Medium expression numbers	Expression			
		High	Medium	Low	Not detected
glioma	11/12 (91.6%)	4	7	0	1
breast cancer	8/12 (66.7%)	1	7	4	0
stomach cancer	8/11 (66.7%)	4	4	3	0
pancreatic cancer	11/12 (91.6%)	8	3	0	1
renal cancer	7/12 (58.3%)	1	6	2	3
colorectal cancer	12/12 (100%)	10	2	0	0
prostate cancer	7/11 (63.6%)	1	6	2	2
urothelial cancer	7/11 (63.6%)	2	5	3	1

Figure S2. CD73 protein expression summary

Protein expression profiles of CD73 in glioma, breast cancer, stomach cancer, pancreatic cancer, renal cancer, colorectal cancer, prostate cancer, and urothelial cancer patients were summarized. These data were obtained from the human protein atlas database (https://www.proteinatlas.org/ENSG00000135318-NT5E/pathology).

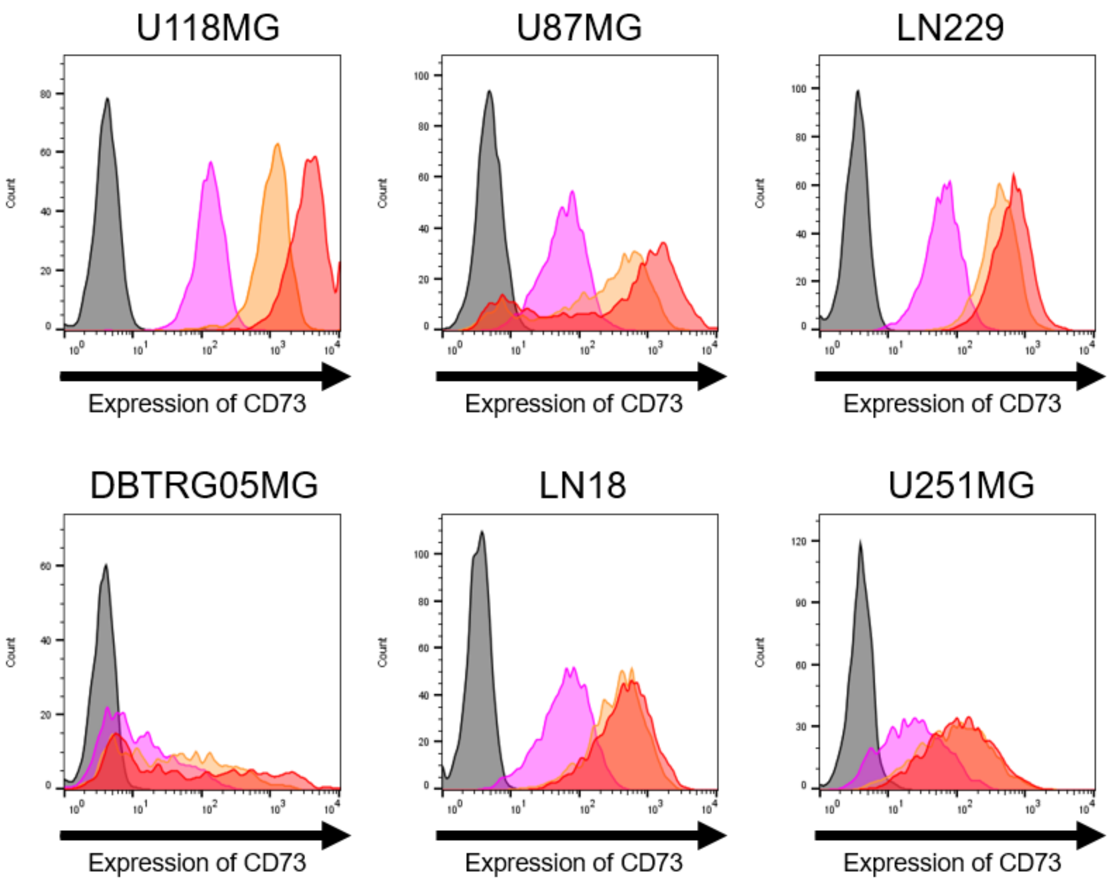

Figure S3. Flow cytometry analysis of CD73 in GB cell lines

The expression of CD73 was evaluated using mAb 0614-5. Red (1 μg/mL), Orange (0.1 μg/mL), Pink (0.01 μg/mL), Black (secondly antibody only, negative control) lines were shown.

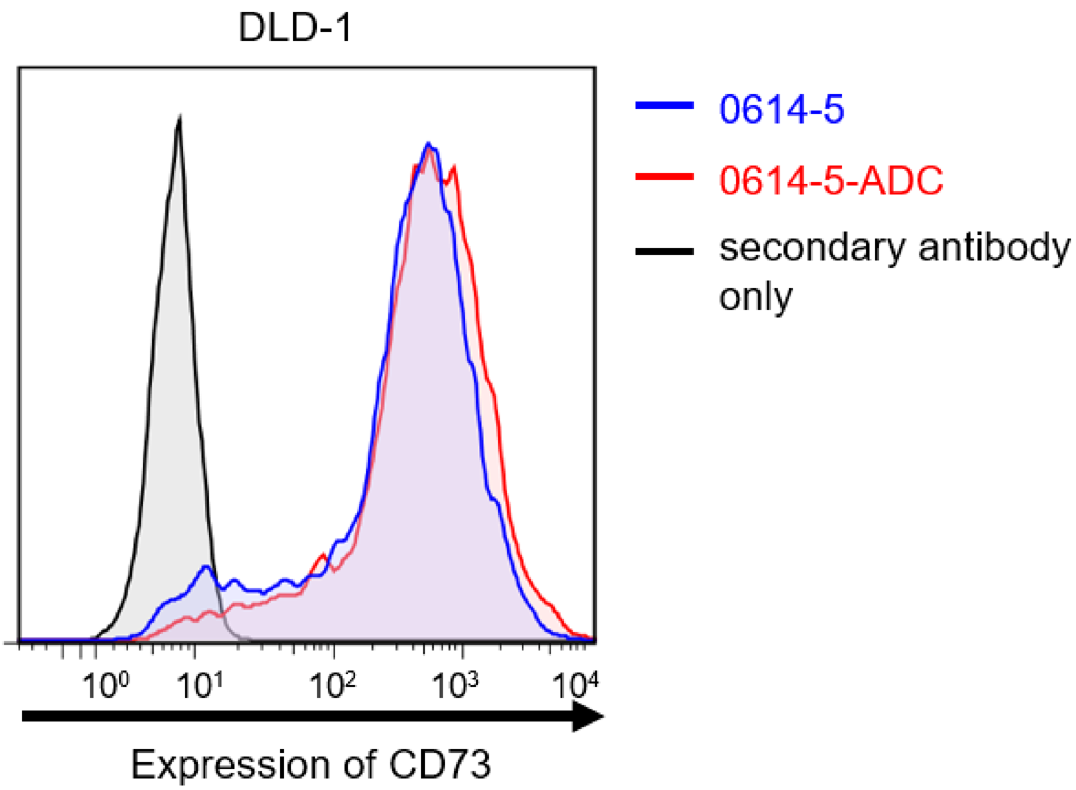

Figure S4. Binding activity of 0614-5 and 0614-5-ADC

Flow cytometry analysis of 0614-5 (blue, 1 µg/mL) and 0614-5-ADC (red, 1 µg/mL) for DLD-1 were shown.

Abbreviations list

ADC: antibody-drug conjugate
AMP: adenosine monophosphate
ATP: adenosine triphosphate
CCLE: Cancer Cell Line Encyclopedia
CRC: colorectal cancer
EC50: Effective Concentration 50
ELISA: Enzyme-linked immunosorbent assays
EPR: Enhanced Permeability and Retention
GB: glioblastoma
IC50: Inhibitory Concentration 50
MMAE: monomethyl auristatin E
NT5E: 5'-nucleotidase ecto
PI: propidium iodide
TCGA: The Cancer Genome Atlas
